# Elevated Expression of AKR1C3 Increases Resistance of Cancer Cells to Ionizing Radiation via Modulation of Oxidative Stress

**DOI:** 10.1371/journal.pone.0111911

**Published:** 2014-11-24

**Authors:** Wei Xiong, Jing Zhao, Hongliang Yu, Xiaoying Li, Shaoqian Sun, Yi Li, Qing Xia, Chuanling Zhang, Qiuchen He, Xianshu Gao, Lihe Zhang, Demin Zhou

**Affiliations:** 1 The State Key Laboratory of Natural and Biomimetic Drugs, Beijing, China; 2 The 1st Affiliated Hospital, Peking University, Beijing, China; 3 School of Pharmaceutical Sciences, Peking University, Beijing, China; 4 Tangshan People's Hospital, Hebei, China; 5 Peking Union Medical College Hospital, Beijing, China; University Health Network, Canada

## Abstract

With the aim to elucidate the etiology of radioresistance, we explored the genetic alterations in non-radioresistant vs. resistant esophageal cancer cells acquired by long-term fractionated radiation. We found AKR1C3, an aldo-keto reductase expressed seldom in most human tissues, expressed higher in radioresistance-acquired cells. Suppression of AKR1C3 via RNAi or its chemical inhibitors restored the sensitivity of the acquired tumor cells and xenograft BALB/c nude mice to ionizing radiation (IR). Cellular monitoring of the oxidative stress in the AKR1C3-elevated cells indicated that IR-induced ROS accumulation and the concomitant DNA damage was significantly alleviated, and such protective consequence disappeared upon AKR1C3 knockdown. These findings uncover the potential involvement of AKR1C3 in removal of cellular ROS and explain, at least partially, the acquired radioresistance by AKR1C3 overexpression. A retrospective analysis of esophageal carcinomas also indicated a significant expression of AKR1C3 in radio-resistant but not radio-sensitive surgical samples. Our study may provide a potential biomarker for predicting prognosis of radiotherapy and even direct a targeted therapy for esophageal cancer and other tumors.

## Introduction

Ionizing radiation (IR) is the most effective nonsurgical treatment for most tumors [1]. Such therapeutic intervention usually relies on the interaction between IR and water molecules in cells that produces excessive reactive oxygen species (ROS) and thus places cancer cells under extensive oxidative stress [2]. The highly unstable ROS react with DNA and other macromolecules in the vicinity, producing gene defect, chromosome aberration and other damage [3]. Mitochondria damage by IR can even lead to prolonged oxidative stress and thus further damages DNA and other cellular molecules [4]. Accumulation of DNA damage is an important factor in triggering IR-induced failure of cell division and proliferation that leads to cell apoptosis [5].

Despite of the therapeutic significance, IR resistance is a major impediment to cancer therapy [6]. It has been reported that repairing of IR-induced DNA damage, either double strand breaks or single strand defects, is one important mechanism underlying cancer recurrence and radioresistance [7]–[11]. Removal of IR-generated ROS by antioxidant enzymes constitutes alternative mechanism leading to radioresistance. Glutathione peroxidase (GPx), peroxiredoxin (TPx) and superoxide dismutase (SOD), the largest class of antioxidant enzymes, have such ability to diminish IR-induced ROS accumulation [12]–[14]. Certain cancer cells and cancer stem cells contain a higher expression of antioxidant enzymes and are thus preferentially spared from irradiation [15], [16]. Discovery of other cellular enzymes involved in counteracting oxidative stress may be helpful for diagnosis of non-susceptible patients to radiotherapy and even direct an effective targeted therapy for malignant tumors.

Esophageal cancer is a common cancer with a 5-year survival rate of 5–19% [17]. There is strong evidence suggesting that radioresistance of esophageal cancer might be congenital, *i.e.* related to gender and ethnicity, or be induced by lifestyle such as smoking [18]. Therefore, esophageal carcinoma may provide an ideal model for identification of cellular factors by which radioresistance is induced. In this study, we found that the acquiring of radioresistance coincided with elevated expression of AKR1C3 in esophageal cancer cells, and suppression of AKR1C3 expression restored the sensitivity of the acquired tumor cells and xenograft animals to ionizing radiation (IR). Furthermore, cellular monitoring of the oxidative stress in the AKR1C3-elevated cells indicated that ROS accumulation and the concomitant DNA damage was significantly alleviated, and such protective consequence disappeared upon AKR1C3 knockdown. A retrospective analysis indicated a significant AKR1C3 expression in radio-resistant but non-resistant surgical esophageal carcinomas. Our findings may provide a new biomarker for prediction of non-susceptible patients to radiotherapy and even direct a targeted therapy for esophageal cancer and other tumors.

## Materials and Methods

### Cell culture and irradiation

KY170 and TE13, two human esophageal squamous cancer cell lines, and their derived radioresistant cell lines KY170R and TE13R were provided as a gift by Department of Radiology, MD Anderson Cancer Center, USA. These cell lines, originally reported by a Japanese group [19], were authenticated and tested by provider using short tandem repeat profiling and karyotyping tests. Cells were passaged for less than 1 month before experimentation. An aliquot of the sub-stock was used for the studies described here. Cells were cultured in high glucose RPMI 1640 (*Invitrogen*) supplemented with 10% fetal bovine serum (*HyClone*) and 50 unit/ml penicillin and 50 µg/ml streptomycin. Cells were maintained in a humidified incubator with 5% CO_2_ at 37°C and split every 3–4 days. Tumor cell irradiation was carried out with a 6MV-X-ray linear accelerator.

### RNAi, AKR1C3-silencing stable cells

AKR1C3-targeting shRNA and scramble-shRNA cassettes were constructed by PCR using up-primer (hU6 promoter): TGGATCCaaggtcgggcaggaagag, down-primer (scramble): AGGATCCAAAAACAACAAGATGAAGAGCACCAACTCGAGTTGGTGCTCTTCATCTTGTTGGGTGTTTCGTCCTTTCGTCCTTT; down-primer (shRNA1): 5′-AGGATCCAAAAACCA GAGGTTCCGAGAAGTAAACTCGAGTTTACTTCTCGGAACCTCTGGCCGGTGTTTCGTCCTTTCCAC-3′, (shRNA2): 5′-AGGATCCAAAAACCTAGACAGAAATCTCCACTACTCG AGTAGTGGAGATTTCTGTCTGGCCGGTGTTTCGTCCTTTCCAC-3′, (shRNA3): 5′-AG GATCCAAAAACTCACTGAAGAAAGCTCAATTCTCGAGAATTGAGCTTTCTTCAGTGAGCCCGGTGTTTCGTCCTTTCCAC-3′. The cassettes obtained were cloned into pSD31 for constitutive expression of shRNA following the method that was prescribed previously [19]. Lentiviral vector production was performed according to Invitrogen's standard protocol. Experiments for lentivector stable transduction were carried out as follows: 1×10^6^ of replicon cells in a T25 flask were seeded and transduced on day 2 in the presence of 8 mg/ml of polybrene. Selection was performed after day 3 by 800 ng/ml of puromycin until the parental cells in parallel experiments completely died.

### Colony formation assay and tumor irradiation

A monolayer of cells at the exponential stage of growth were irradiated using a 6 MV-X-ray linear accelerator at appropriate doses (2, 4, 8 Gy), and the irradiated cells were plated to grow in six-well plates. After incubation for 7-10 days, the surviving cells were stained with crystal violet and then counted. Each experiment was carried out in triplicate and repeated twice. Tumor irradiation was performed as follows: when the diameter of xenograft tumors reached 6–8 mm, the hind limbs of xenograft mice were irradiated with a single dose (15 Gy). After irradiation the tumor growth was monitored for up to 36 days.

### RNA extraction and Microarray

The RNA was extracted from each cultured cell line using SV Total RNA Isolation Kit (*Promega*) according to the manufacturer's protocol. The extracted RNA was then treated with RNase-Free DNase set (*QIAGEN*) to remove any contaminating genomic DNA according to the manufacture's protocol. Gene profiling, in triplicate, was carried out by Illumina Shanghai Corporation using a microarray of Illumine Human-6 V3 and data were collected and analyzed with the Illuminated Beadstudio Application software (GSE61620, GSE61772 and GSE61816). Genes with an Illumina DifferScore of ≥20 were considered to represent up-regulation, whereas those with an Illumina DifferScore of ≤−20 were considered to be down-regulation. P<0.05 indicated a significant difference.

### Real-time RT-PCR

The isolated total RNA was quantified with a UV spectrophotometer (*Eppendorf*) and then 1.0 µg of RNA was reverse transcribed using First Strand cDNA Synthesis Kit (*TOYOBO,Japan*) with random primers in a reaction volume of 20 µl. The PCR reaction was carried out using Go Taq qPCR Master Mix kit (*Promega*) according to the protocol provided by the manufacturer and the cDNA was then used as a template for detection of the different gene expressions. PCR primers were designed by Primer 3.0 and a blast search to check their specificity. Primers for PCR amplification of AKR1C3 were 5′ATTTGGCACCTATGCACCTC 3′ (upstream) and 5′TGAGTTTTCCAAGGCTGGTC 3′ (downstream) and β-acting 5′ AGCGAGC ATCCCCCAAAGTT 3′ (upstream) and 5′GGGCACGAAGGCTCATCATT 3′ (downstream). The relative mRNA levels of the different genes were calculated as follows: ΔCT (sample)  =  CT (gene) - CT (GAPDH); ΔΔCT  =  ΔCT (post-irradiation time point) - ΔCT (0 h); Relative expression  = 2^-ΔΔCT^. Each RT-PCR was repeated at least twice.

### Western blot assay

Cultured Cells were washed once with PBS buffer then lysed in lysis buffer (1% SDS, 50 mM Tris, pH 7.4, 0.15 M NaCl, 1 mM NaF, 10 mM phenylmethylsulfonyl fluoride, 1 mM sodium orthovanadate, 1 mM EDTA) for 5 min and passed through a 27-gauge needle. Lysates were centrifuged at 12,000 *g* for 1 min and protein concentrations were determined using a Bio-Rad DC protein assay. Equal amounts of protein were separated by 4∼20% SDS-PAGE and transferred to nitrocellulose membranes. Membranes were blocked with 5% skim milk or 3% bovine serum albumin in TBST (10 mM Tris, pH 7.5, 150 mM NaCl, 0.1% Tween 20) for 1 h. Primary and secondary antibodies were used according to the manufacturer's instructions, and this was followed by detection with an enhanced chemiluminescence technique (*Amersham Biosciences*). Western quantitations were performed as follows: Western blots were scanned by a densitometer to obtain the density of each band. Then, the ratio of the density of a band of interest to that of its internal control band (GAPDH) was relative to the ratio from the negative control experiments. Each Western blotting was repeated at least twice.

### Overexpression of AKR1C3

Human AKR1C3 cDNA was purchased (*Origene*, Cat. No: SC321532), cloned into pcDNA3.1 and then transfected into KY170 cells according to the protocol provided.

### Flow cytometry assays

Ethanol-fixed cells were treated with RNase A (0.1 mg/ml) for 30 min followed by incubation with PI (35 µg/ml). DNA content of PI-stained cells was analyzed by FACScan (Becton Dickinson) and the percentage of cells with G1, S, and G2/M DNA content was calculated using MODFIT software. Flow cytometry measurements of dihydroethidium (DHE)-fluorescence were used to measure cellular ROS levels. Monolayer cultures were incubated in 10 µM DHE for 45 min and then harvested. DHE-fluorescence was analyzed by flow cytometry (excitation wavelength 488 nm, emission 585 nm). The mean fluorescence intensity (MFI) was calculated after correction for autofluorescence and the fold change was calculated relative to scramble-KY170R cells. Each flow cytometry assay was repeated twice.

### Animal experiments

Male BALB/c nude mice (SPF, purchased from Vital River Laboratories, Beijing, China), aged 4–6 weeks, were housed with an inverse 12 hours day-night cycle with lights on at 8:30pm in a temperature (22±1°C) and humidity (55±5%) controlled room. All mice were given water and chow *ad libitum* at all times. Xenograft nude mice were generated as follows: KY170R-shRNA1 and KY170R-scramble cells were cultured in complete medium to 70% to 80% density. After trypsinizing, cells were washed once with PBS, and counted. The male, 4-week-old to 6-week-old nude mice were randomized into two group and injected in the upper portion of a hind limb with a density of 2*10^6^/100 µl of KY170R-shRNA1 and KY170R-scramble cells, respectively. Tumor growth was monitored daily and when the tumor diameter reached 6 to 8 mm, the “irradiation group” animals were irradiated once with a dose of 15 Gy. The regression in orthotropic tumor growth was followed for up to 36 days, the tumor volume being calculated as formula V = π/6 (a*b^2^), where a is the longest and b is the shorter perpendicular tumor axis. Tumor volume was measured until the tumors reached a volume of about 1.0 cm^3^. Then the mice were sacrificed by cervical dislocation for subsequent experiments.

### Immunohistochemistry of tissue sections

Immunohistochemistry of human esophageal cancer tissue sections, acquired from pathology department of Peking University First Hospital, and xenograft tumors was performed as follows: 4–6 µm tissue sections were mounted and heated at 72°C for 30 min. Sections were deparaffinized with xylene, rehydrated in graded EtOH and rinsed with 0.1 M Tris-HCl (pH 7.6). Endogenous peroxidase activity was blocked by incubating the tissue sections with 3% H_2_O_2_ for 30 min. Antigen retrieval was performed with 0.01 M sodium citric acid buffer (pH 6.0) at 95°C for 1 h. Non-specific binding was blocked by incubation of the tissue sections with 0.1 M Tris-HCl containing 10% goat serum for 2 h. AKR1C3 was then determined by incubating the sections with mouse anti-AKR1C3 monoclonal antibody at a 1∶200 dilution in the above blocking solution in a moist chamber at 4°C overnight. After washes with 0.1 M Tris-HCl, the sections were treated with 1∶400 dilution of biotinylated horse anti-mouse secondary antibody and incubated at room temperature for 2 h. After a further rinse with 0.1 M Tris-HCl, antibody binding was detected by incubating the tissue sections with HRP-conjugated streptavidin at room temperature for 30 min. DAB-H_2_O_2_ substrate was then added to the slides which were incubated at room temperature for an additional 4 min. Tissue sections were stained with hematoxylin, dehydrated in graded alcohol, cleared in xylene, and mounted with Permount Mounting Media for visualization by light-microscopy.

### Statistical analysis

The Student t-Test was used to determine the statistical difference between means of two groups and data. P values <0.05 were considered significant.

### Ethics Statement

The animal study was carried out in strict accordance with the recommendations in the Guide for the Care and Use of Laboratory Animals of the National Institutes of Health. All efforts were made to minimize suffering. And written informed consents were obtained from all patients or their families for research using these tissue samples. All the cell, animal experiments and Immunohistochemistry of tissue sections study have been approved by the Peking University Institutional Review Board (Certificate No. IRB00001052-12037).

## Results

### The differentiated expressions of AKR1C3 in cancer cells

**Figure 1:**
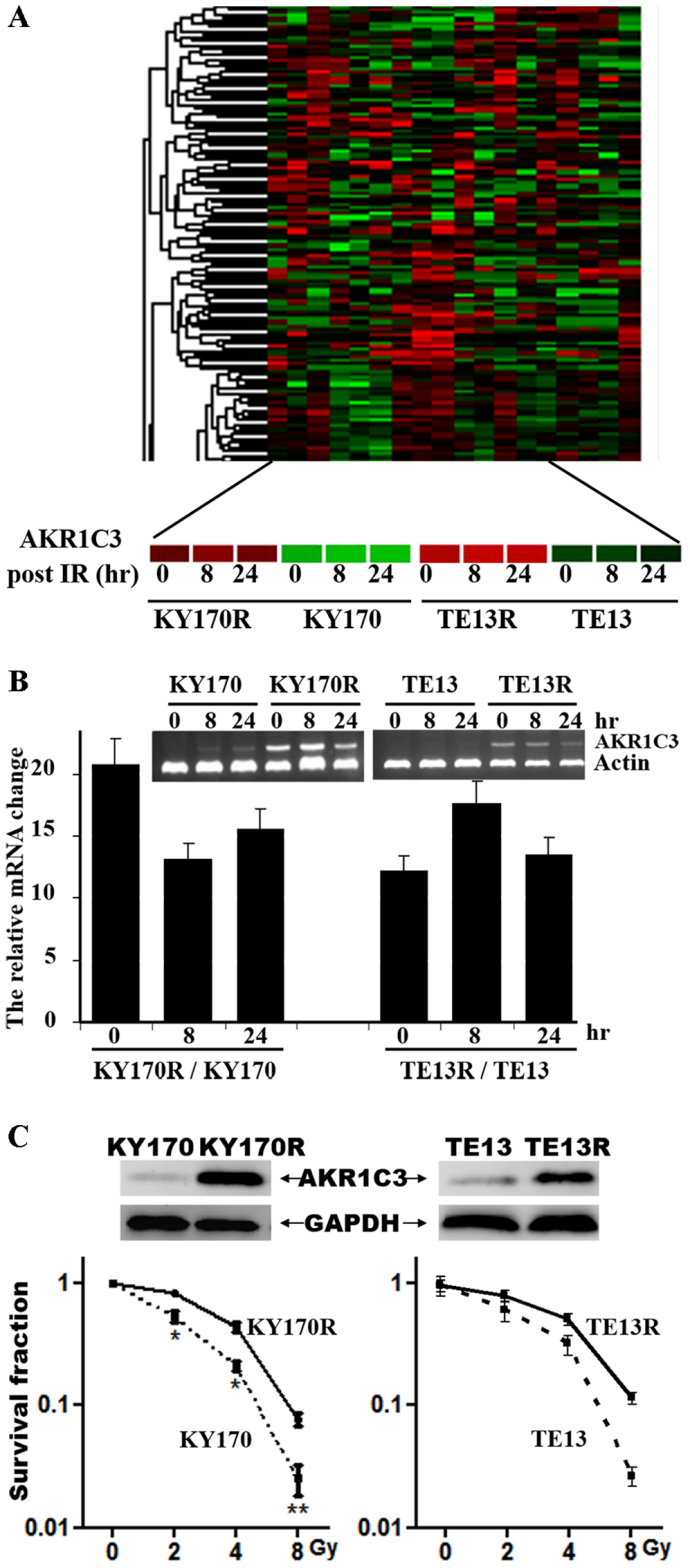
Profiling of AKR1C3 in radio-resistant and -sensitive cancer cells. (**A**) Genome-wide profiling of gene expression in radioresistant KY170R and TE13R *versus* radiosensitive KY170 and TE13, respectively, at 0, 8 h and 24 h after irradiation. (**B**) Validation of the elevated expression of AKR1C3 at the mRNA level in KY170R vs. KY170 and TE13R vs. TE13 cells prior to and 8 and 24 hr after irradiation as determined by RT-PCR. (**C**) Validation of elevated expression of AKR1C3 in radioresistant KY170R determined by Western blot and the sensitivity of KY170 and KY170R cells to irradiation as determined by a colony-formation assay.

To elucidate the etiology of radioresistance, two radioresistant esophageal tumor clones, KY170R and TE13R, have been established by continuous fractionated irradiation of their parental cells, KY170 and TE13 [20]. Genome-wide profiling of gene expression in KY170R *v.* KY170 and TE13R *v.* TE13 using Illumine Human-6 V3 microarray indicated that over 900 genes were found to be remarkably differentiated (**Fig. 1A**), consistent with previous report [21]. Among them, AKR1C3, an aldo-keto reductase existing at a low level in most human tissues, attracted our attention due to its significant expression in both radioresistant cells. Real time RT-PCR (**Fig. 1B**) and Western blotting (**Fig. 1C**) analyses confirmed the elevated expression of AKR1C3 in KY170R and TE13R cells, respectively, as compared with their parental cells: ∼10-fold at the mRNA level and much higher at the protein level. This profile revealed the coincidence of elevated expression of AKR1C3 and radioresistance in esophageal KY170R and TE13R cancer cells. Colony-formation assays verified that KY170R and TE13R were indeed more resistant to IR than their parental KY170 and TE13 cells, with the estimated dose for reduction of survival by 90% for KY170 vs. KY170R as 5.5 Gy vs. 7.8 Gy (**Fig. 1C**).

### The effect of AKR1C3 on the response of tumor cells to irradiation

**Figure 2:**
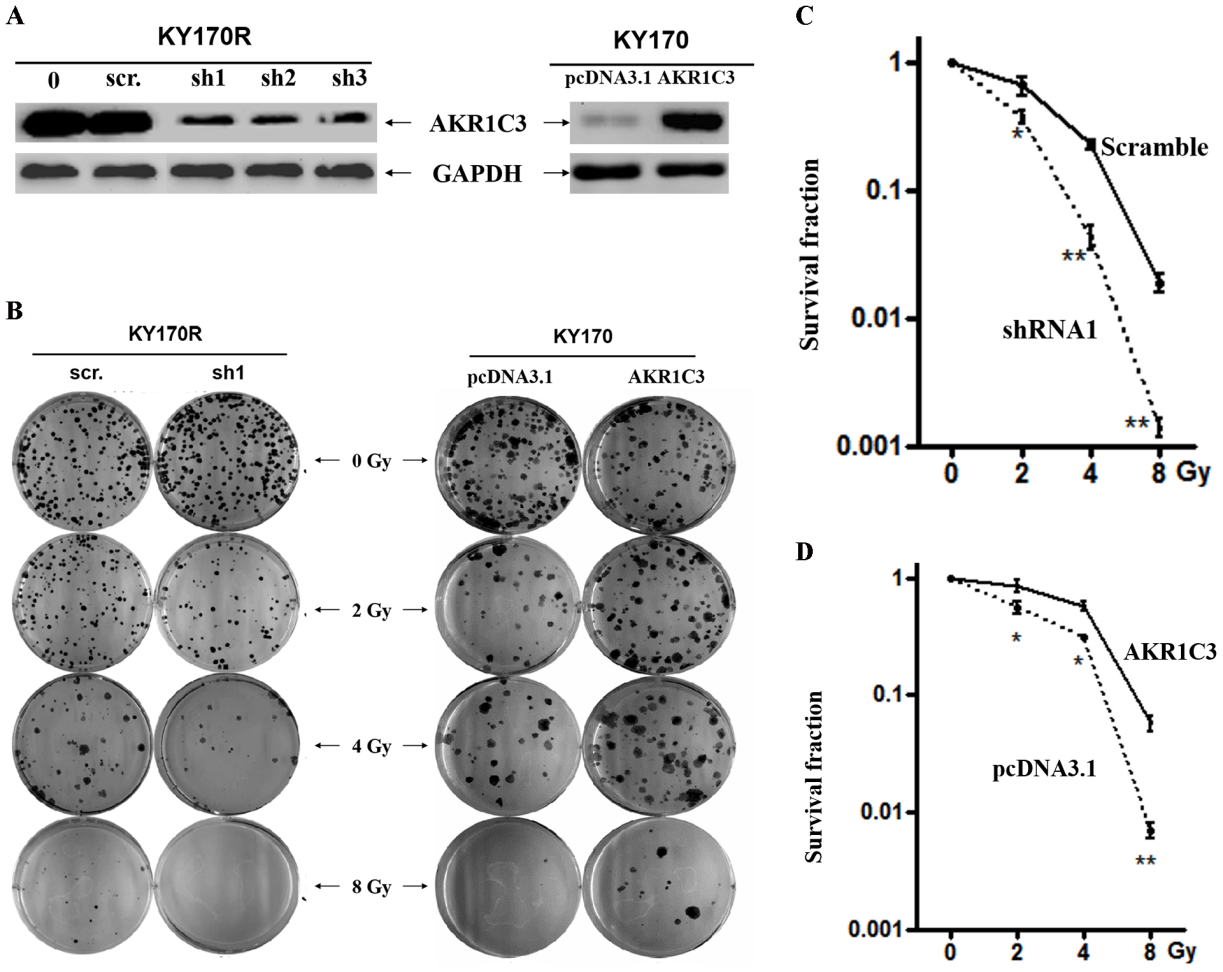
Cell-based evaluation of the role of AKR1C3 on the response of esophageal cancer cells to irradiation. (**A**) Representative observations of stable knockdown of AKR1C3 in KY170R cells by three independent shRNAs and overexpression of AKR1C3 in KY170 cells. (**B**) The effects of AKR1C3 knockdown in KY170R cells and AKR1C3 overexpression in KY170 on the outcome of irradiation, dosed from 0 to 8 Gy, determined by colony-formation assay. A scramble shRNA (scr.) and the empty pcDNA3.1 vector acted as a negative control, respectively. (**C**) The survival curves for comparisons of the effect of AKR1C3 on the sensitivity of KY170R cells (knockdown) and (**D**) KY170 cells (overexpression) to irradiation, as determined by colony-formation assays.

Given the significant difference in expression of AKR1C3 between resistant and non-resistant esophageal cancer cells, we questioned whether it might be a primary cause or merely a downstream consequence of radioresistance. To examine this issue, the expression levels of AKR1C3 in KY170R and TE13R were down-regulated by shRNAs delivered by lentivector pSD31 and the effects of AKR1C3 knockdown on radioresistance were characterized. To exclude off-target issues, three shRNAs were utilized in parallel to elucidate the impact of RNAi on KY170R cells with a scramble shRNA as a negative control. Western blotting analysis demonstrated that all three shRNAs used in this study exerted strong potency in transduced KY170R cells with>80% AKR1C3 being down-regulated as compared with the scramble-shRNA (**Fig. 2A**). Characterization of AKR1C3 knockdown on cell proliferation indicated that KY170R-shRNA1, KY170R-shRNA2 and KY170R-shRNA3 (three stable cell lines generated by pSD31-mediated transduction) proliferated at almost the same rate as KY170R-scramble cell, which itself had a rate almost identical to that seen in KY170R and KY170 cells (**Fig. S1A in File S1**). This suggests that AKR1C3 is not a required gene for proliferation of these cancer cells. By contrast, irradiation disclosed the biological effect of AKR1C3 on promoting cell survival. Colony-formation assays indicated that the transduced KY170R-shRNA1, -shRNA2 or -shRNA3 cells became substantially more sensitive to radiation with fewer clones formed than KY170R-scramble cells (**Figs. 2B–C** and **Fig. S1B in File S1**), which had an estimated dose of 6.5 Gy for reduction of survival by 90%, much higher than KY170R-shRNA1 cells (∼4.5 Gy).

### Confirmation of AKR1C3 effect on the response of tumor cells to irradiation

In order to confirm the protective effect of AKR1C3 disclosed by RNAi, the radioresistant KY170R and its parental KY170 cells were cultured in the presence of 200 µM methyl jasmonate (MJ) and then irradiated. MJ is a well-established small molecule with its structure resembling to prostaglandins, the substrate of aldo-keto reductases, and thus acts as a competitive inhibitor of AKR1C3. MeJ displays great potency in the cellular system due to the increased cellular uptake with its IC_50_ for AKR1C3 at ∼150 µM [22]. It was found that treatment with MeJ also substantially enhanced the response of KY170R cells to irradiation and such MJ-mediated enhancement was not observed in KY170 cells (**Fig. S1C in File S1**). It appeared that suppression of AKR1C3 activity via its chemical inhibitor could also sensitize KY170R cells to IR. Furthermore, we examined whether elevated expression of AKR1C3 alone might cause KY170 cells to be resistant to irradiation. An AKR1C3 expression construct, pcDNA3.1-AKR1C3, was introduced into parental KY170 cells to generate AKR1C3-overexpressing transfectants (**Fig. 2A**). It was found that overexpression of AKR1C3 in KY170 cells, about a 5-fold increase, allowed more colonies to survive than in the case of pcDNA3.1-transfected cells upon the same dose of irradiation (**Figs. 2B and 2D**). Clearly, it is the elevated expression of AKR1C3 that renders both KY170R and TE13R cells substantially resistant to radiation.

### The impact of AKR1C3 on the growth of irradiated xenograft tumors

**Figure 3:**
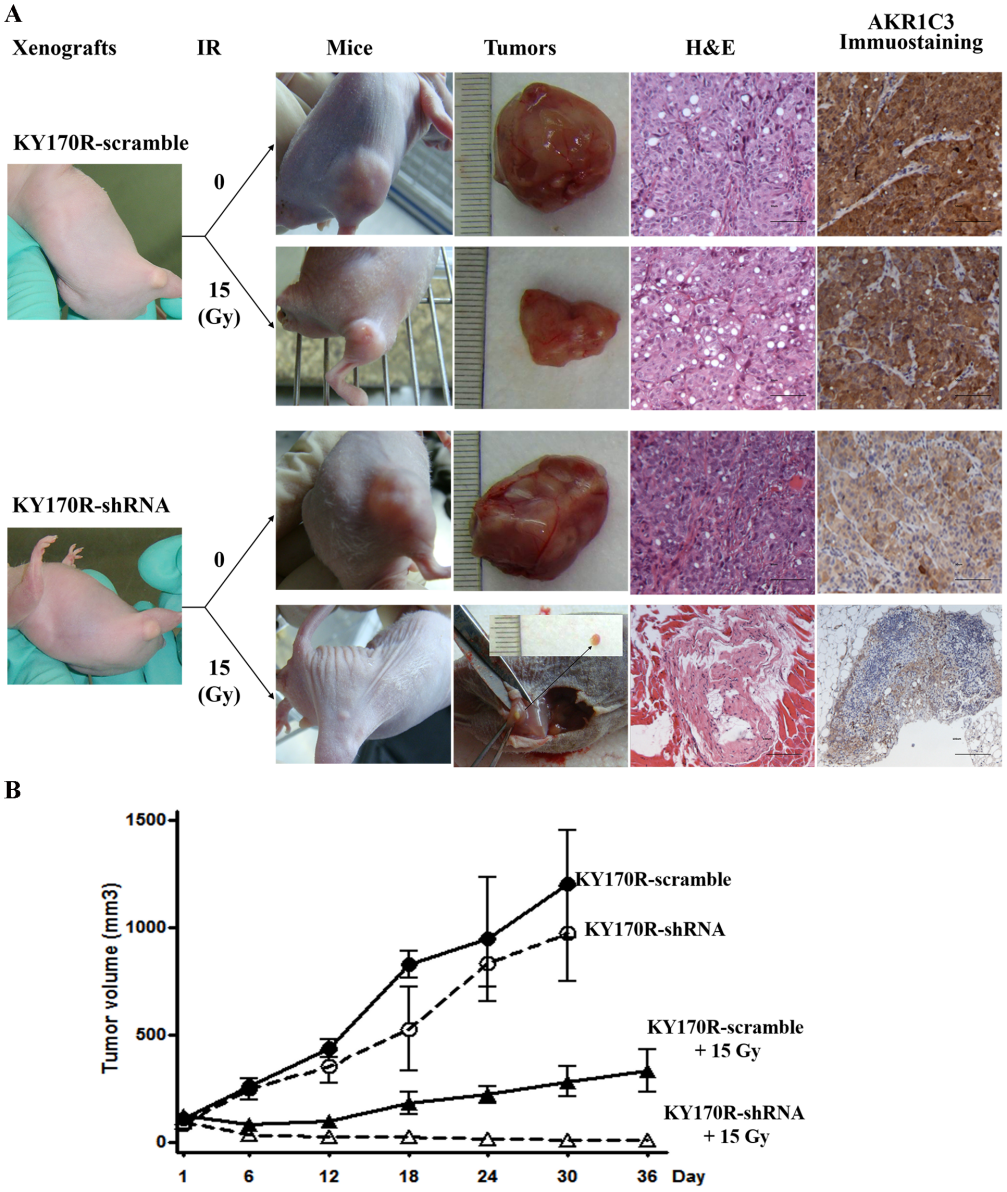
Xenograft-based evaluation of AKR1C3 elevation on the response of esophageal cancers to irradiation. (**A**) Representative observations of the growth of xenograft KY170R-scramble and KY170R-shRNA1 tumors, and the responses of these tumors to a single dose (15 Gy) of irradiation in terms of tumor volume, HE staining and immunochemical staining of AKR1C3. (**B**) The time course of growth of KY170R-scramble and KY170R-shRNA1 xenograft tumors with or without a local IR treatment.

We then explored whether AKR1C3-mediated cellular radioresistance was also operative in xenograft tumors. Two xenograft models were established by subcutaneously implanting KY170R-shRNA1 and KY170R-scramble cells, separately, in nude mice. We found that both xenotransplant tumors grew fast with the former growing just slightly slower than the latter (**Figs. 3A and B**), supporting the cell-based observation that AKR1C3 has far less effect on tumor growth. When the tumors had reached an average volume of approximately 100 mm^3^ (**Fig. 3A**), 20 xenograft mice from each type were selected, half of them were subjected to local radiation at a single dose of 15 Gy and half remained untreated. We found all xenografted tumors in 10 irradiated KY170R-shRNA1 mice were sensitive to IR treatment with 8 mice becoming almost tumor-free 5 weeks post irradiation (**Figs. 3A–B** and **Fig. S2 in File S1**). Staining with hematoxylin and eosin (HE) revealed the absence of neoplasm but only a tiny shrunken residue in the irradiated xenotransplants (**Fig. 3A**). By contrast, the non-irradiated xenograft tumors in KY170R-shRNA1 and KY170R-scramble mice grew to an average volume of 1000 mm^3^ with full proliferating cells (**Figs. 3A–B** and **Fig. S2 in File S1**). In the case of the mice with KY170R-scramble xenotransplants, irradiation led to a poor outcome: tumor growth was initially repressed upon irradiation but resumed within two weeks (**Fig. 3B**); extensive neoplasm and strong AKR1C3 expression was detected in the tumor tissues (**Fig. 3A**). These results disclosed the effect of AKR1C3 elevation in protecting xenograft tumors from irradiation, confirming the hypothesis derived from the cell-based experiments.

### Mechanistic elucidation of AKR1C3-mediated radioresistance

**Figure 4:**
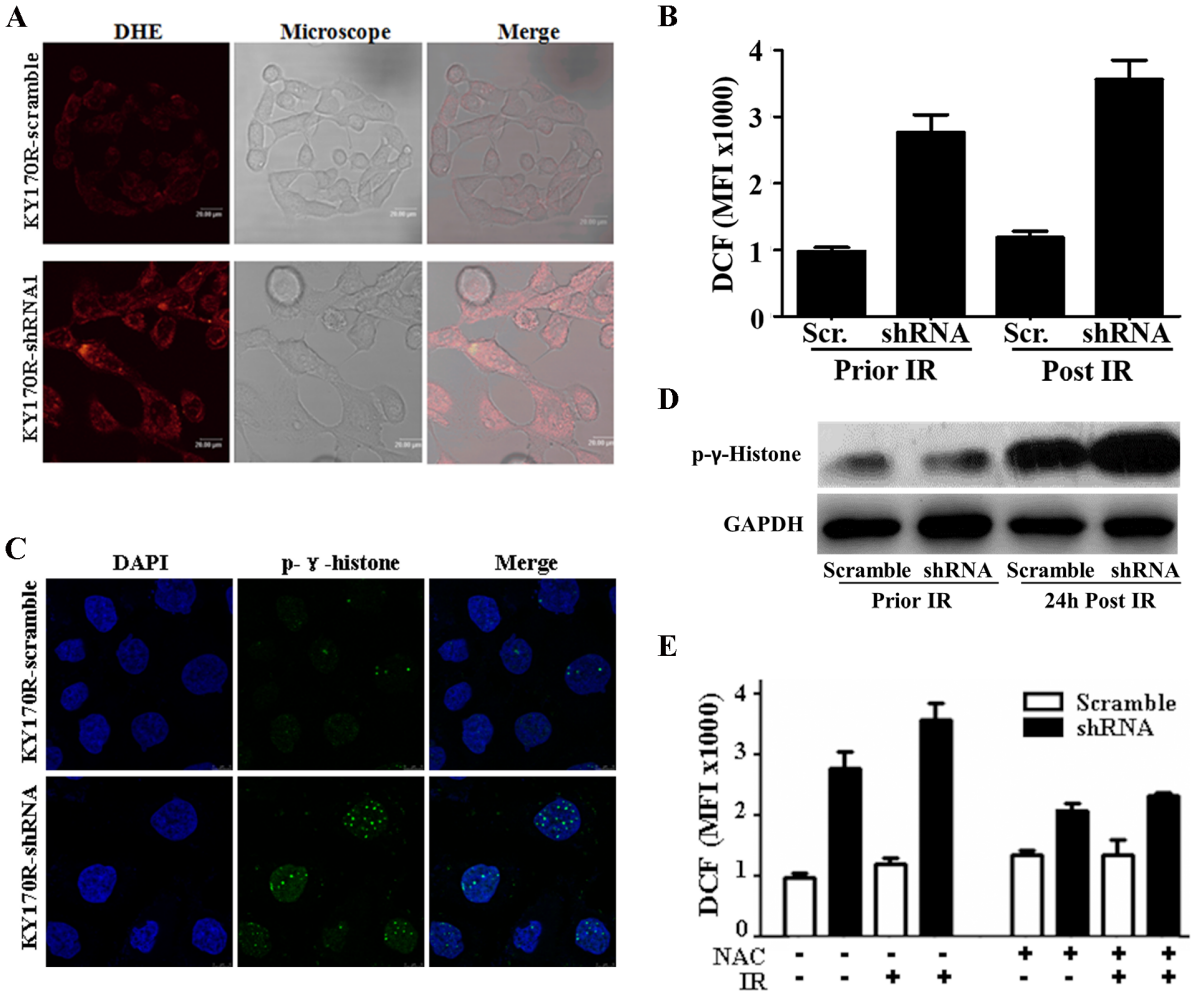
Mechanistic exploration of AKR1C3 as a cellular factor for protecting cells from irradiation damage. (**A**) Representative microscopic view of the accumulation of ROS in KY170R-scramble *v.* KY170R-shRNA1 cells stained by DHE. (**B**) Quantifications of the ROS levels in KY170R-scramble *v.* KY170R-shRNA1 cells prior to and 24 h after IR at a dose of 4 Gy. (**C**) Representative microscopic view of phospho-γ-histone foci in KY170R-scramble *v.* KY170R-shRNA1 cells. (**D**) Characterizations of phospho-γ-histone in tested cells by Western blotting. (**E**) N-Acetyl cysteine (NAC)-mediated scavenging of ROS at a IR dose of 4 Gy. Each quantitative measurement was carried out in triplicate and repeated at least twice with an error bar less than 25%.

To explore the mechanism by which AKR1C3 exerts its effect on the outcome of IR, we compared the levels of cellular ROS and DNA damage, the critical mediators and the immediate consequence of IR-induced cell death [23], in KY170R-shRNA1 versus KY170R-scramble cells. It was found that prior to irradiation approximately 2-fold less ROS was in KY170R-scramble than in KY170R-shRNA1 cells (**Figs. 4A–B**). Upon irradiation, the level of ROS remained low in KY170R-scramble cells but remarkably increased in KY170R-shRNA1 cells with the difference being almost 3 folds (**Fig. 4B**). Coincident with simultaneous AKR1C3 knockdown and ROS increase in irradiated KY170R-shRNA cells, the number of foci of phospho-γ-histone (**Figs. 4C–D**), a biomarker of DNA damage [24], accompanied by many more chromosomal breaks and gross nuclear abnormalities (**Figs. S3A–B in File S1**), was much higher than in irradiated KY170R-scramble cells 48 h post irradiation. A parallel experiment performed in the presence of N-acetyl cysteine (NAC), a chemical scavenger of ROS [25], indicated that the IR-induced DNA damage in KY170R-shRNA1 cells and the derived morphologic features were prevented (**Fig. 4E**). We concluded that AKR1C3 has the same ability as NAC to alleviate oxidative stress and thus diminish radiation-induced DNA damage, conferring radioresistance.

### Retrospective studies of radioresistance and AKR1C3 expression in esophageal carcinomas

We performed retrospective studies to assess the potential relevance of AKR1C3-related radioresistance in esophageal carcinomas. Esophageal tumor samples, a small piece of tumor specimens taken for pathological diagnosis, were collected from 28 patients who were unable or unwilling to accept surgery (**Table S1 in File S1**). These tumor specimens were then screened via immunohistochemistry to determine the expression levels of AKR1C3. All patients accepted esophageal bed area conformal radiation therapy with the radiation dose at 58–64 Gy. The curative effects were evaluated by reviewing chest CT one months after radiotherapy. Of these patients, 20 were congenitally resistant with tumor volumes shrank less than 50% upon IR; 8 were radiosensitive and became almost tumor-free upon IR treatment.

**Figure 5:**
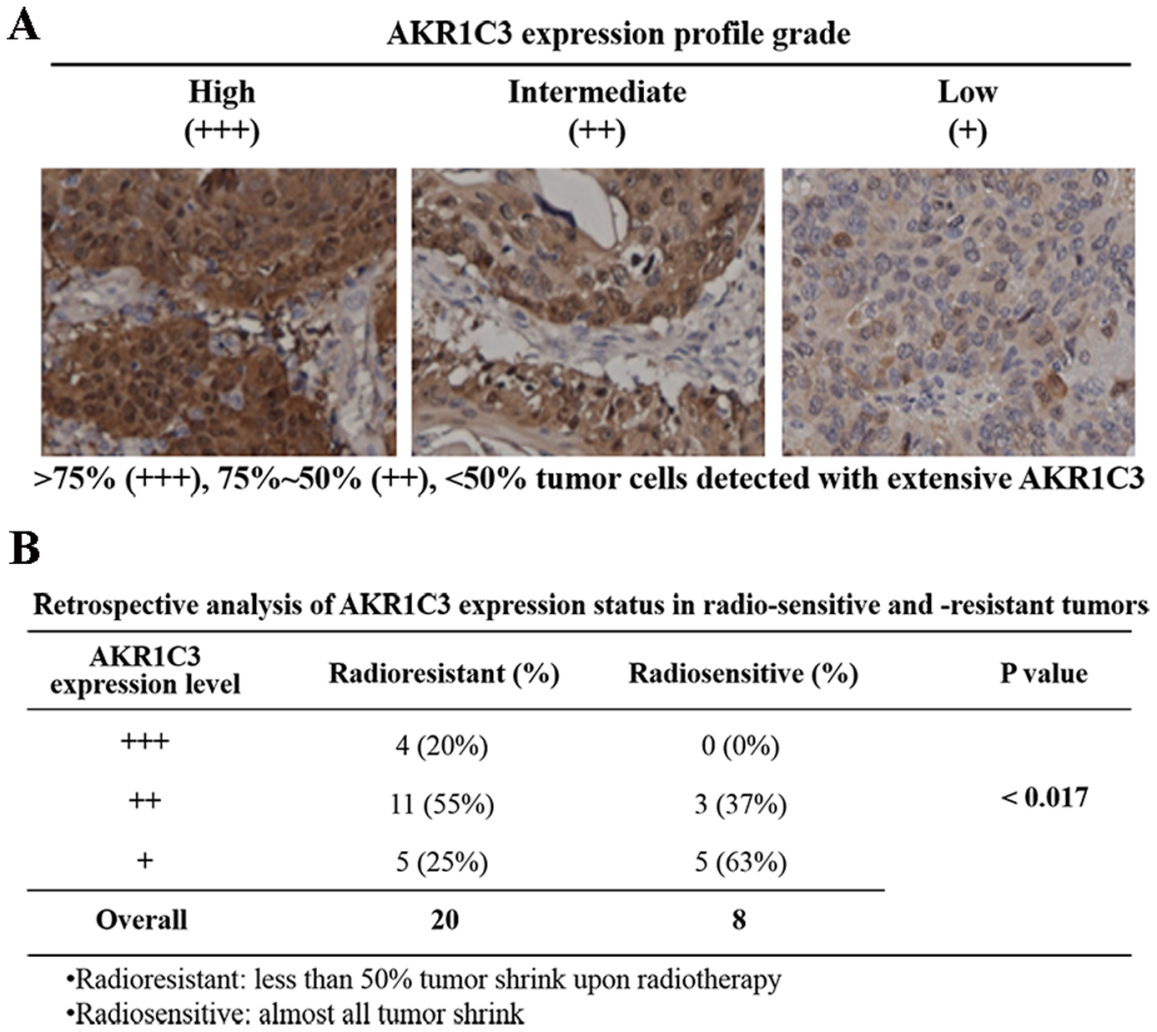
Retrospective analysis of the relevance of AKR1C3 expression and the outcome of IR in esophageal carcinomas. (**A**) Subjective levels of expression of AKR1C3, namely, high (+++), intermediate (++), low or none (+/−), in esophageal carcinomas. (**B**) Levels of expression of AKR1C3 in esophageal carcinoma from 28 patients who were congenitally sensitive or resistant to IR.

Immunohistochemical staining analyses indicated that 7 (35%) of the radioresistant tumor specimens displayed high levels (+++, *i.e.*>75% tumor cells are AKR1C3 positive), and 8 (40%) displayed intermediate levels of AKR1C3 expression (++, *i.e.* 75%∼50% tumor cells contains AKR1C3 protein) (**Fig. 5A–B** and **Fig. S4 in File S1**). The remaining 5 (25%) had a low or undetectable levels of AKR1C3 expression (+/−, *i.e.* 0–50% tumor cells were AKR1C3 positive). By contrast, none of the 8 radiosensitive tumor samples were found to express a high level (+++) of AKR1C3. Only 3 (37%) of the radiosensitive tumor samples had intermediate levels of AKR1C3 but 5 (63%) showed a low or undetectable level (+/−) of AKR1C3 expression (**Fig. 5A–B** and **Fig. S4 in File S1**). Clearly, a strong correlation (p <0.017) exists between elevated expression of AKR1C3 and radioresistance of esophageal carcinoma, implying that AKR1C3 might be a prognostic marker for cancer radiotherapy.

## Discussion

ROS are the key mediators of cellular oxidative stress which determines the cellular redox environment [26]. Excessive levels of ROS are critical in IR-induced cell death. Irradiated cells, in particular those under intermittent exposure that generate excessive ROS, will exist in a state of oxidative siege in which survival requires cells to empower their defense systems to escape oxidative damage. It has been demonstrated that superoxide dismutase constitutes the first line of antioxidant defense systems - catalyzes the breakdown of the superoxide into hydrogen peroxides, which are then removed by catalase [23]. Elevated expressions of superoxide dismutase 1 (SOD1) have been reported in human gliomas and thus conferred radioresistance [15], [16]. Glutathione peroxidase and methionine reductase are also antioxidant enzymes which metabolize ROS [23].

**Figure 6:**
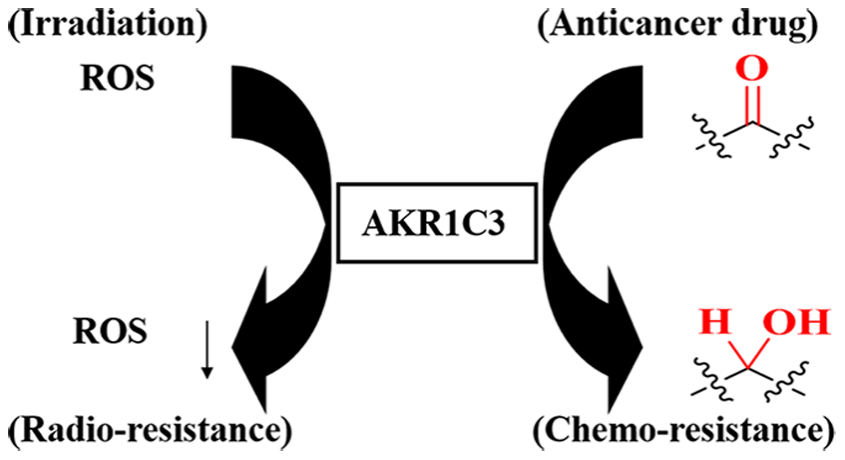
A proposed mechanism of AKR1C3-mediated radioresistance and certain cases of chemo-resistance *via* its reductive capability.

In this study, we reported the coincidence of differentiation of AKR1C3 expression, cellular ROS level and DNA damage in radioresistant KY170R vs. non-resistant KY170 cells. We propose that AKR1C3 is a cellular component participating in the process of scavenging of cellular ROS, sharing the same properties as antioxidant enzymes, at least in *in vivo* to escape oxidative damage (**Fig. 6**). Removal of ROS, either by antioxidant enzymes, AKR1C3 or other unidentified cellular factors, might be a common mechanism account for radioresistance. Unlike glutathione peroxidase (GPx), peroxiredoxin (TPx) and superoxide dismutase (SOD) which are found in essentially all living organisms, AKR1C3 is expressed at a relatively low level in most normal tissues except the prostate and mammary gland [27], [28]. Thus, the expression level of AKR1C3 in tumor cells might be useful as a biomarker for prediction of cellular radioresistance.

AKR1C3 has received extensive attention due to its strong reductive activity towards a variety of substrates. It catalyzes the NADPH-dependent reduction of endogenous aldehydes and ketones to the corresponding alcohols [28], [29]. It also catalyzes the conversion of prostaglandin D_2_ (PGD2) to 11β-PGF2, PGH2 to PGF2a and quinone to catechol [30]. The elevated expression of AKR1C3 in tumor cells will maintain a reductive cellular environment, which not only leads to lower concentrations of ROS but may also be associated with chemoresistance. AKR1C3 has the capability of reducing carbonyl-containing anticancer drugs, such as doxorubicin, into the related alcohols thereby destroying their anticancer effect [31]. Such trend is also observed in KY170R cells which displayed chemoresistance towards doxorubicin as compared to KY170 cells (**Fig. S3C in File S1**). Other groups have demonstrated that elevated expression of AKR1C3 can suppress differentiation of HL-60 tumor cells [28], [32] and promote angiogenesis of PC-3 tumor cells [33], both associated with a poor therapeutic outcome [34]. It is possible that cellular redox-directed intervention *via* AKR1C3 might, to some extent, overcome radio- and chemo-resistance (**Fig. 6**) and even promote the differentiation of tumor cells, offering a new strategy for the treatment of malignancy.

The close relevance of AKR1C3 and radioresistance observed in esophageal carcinomas might have significant impact on cancer target therapy. Elevated expression of AKR1C3 has been frequently found in human malignancies, such as renal carcinoma [35], prostate cancer [36] and breast cancer [29]. A very recent survey of the expression of AKR1C3 in 2490 surgical samples from 19 types of cancer revealed a strikingly elevated expression of AKR1C3 in a subset of hepatocellular (58%); bladder and gastric (>50%); and non-small cell lung (48%) carcinomas [37]. Interestingly, it was found that elevated expression of AKR1C3 in lung cancer was restricted to non-small cell lung cancer; no AKRIC3 expression was detected in small cell lung carcinomas (SCLC) [37]. These observations correlate surprisingly well with clinical observations: SCLC is always highly radiosensitive and radiotherapy can significantly enhance survival of SCLC patients [38]. In addition, breast cancer patients with higher levels of expression of AKR1C3 had a worse overall prognosis [39] after irradiation and a higher rate of late recurrence [40]. These clinical observations suggest that elevated levels of expression of AKR1C3 lead to radioresistance in tumors other than esophageal cancer. It also suggests that a combination of IR and suppression of AKR1C3 represent an effective way to eradicate malignant tumors.

Collectively, we explored the non-radioresistant vs. resistant esophageal cancer cells and found the coincidence of differentiation of AKR1C3 expression, cellular ROS level/DNA damage and acquired radioresistance. AKR1C3 acts as a switch controlling the response of tumor cells and the derived xenografts to IR. Cellular monitoring of the oxidative stress in the AKR1C3-elevated cells indicated that IR-induced ROS accumulation and the concomitant DNA damage was significantly alleviated, and such protective consequence disappeared upon AKR1C3 knockdown. A retrospective analysis of esophageal carcinomas also indicated the significant trend that a higher expression of AKR1C3 was detected in radio-resistant but not radio-sensitive surgical samples. Our study suggests that AKR1C3 might share the same properties as antioxidant enzymes in alleviation of ROS accumulation in tumor cells and may serve as a potential biomarker for predicting prognosis of radiotherapy and even direct a targeted therapy for esophageal cancer and other tumors.

## Supporting Information

File S1 **Supporting information.** Figure S1, Characterization of AKR1C3 expression and effect on cell proliferation. (**A**) Comparisons of cell proliferation, based on AlamarBlue assay, among KY170R-shRNA1, -shRNA2, -shRNA3 and –scramble cells together with their parental KY170R and KY170 cells to explore the effect of AKR1C3 knockdown on the growth of cells. Each experiment was carried out at least in triplicate and repeated twice with the error bar less than 20%. (**B**) Survival curves for comparison of the effect of AKR1C3 knockdown (sh1, sh2 and sh3 vs. scr.) and (**C**) overexpression (AKR1C3 vs. pcDNA3.1) on the sensitivity of KY170R, KY170 cells and TE13R cells to irradiation, which were determined by colony-formation assay. Each experiment was carried out at least in triplicate and repeated twice. Figure S2, Representative scale-up analyses of the effect of AKR1C3 on irradiated tumor stained by immunochemical and HE staining. Figure S3, Mechanistic exploration of AKR1C3 involvement in radioresistance. (**A**) Representative microscopic view and (**B**) quantitative comparisons of nuclear abnormalities in KY170R-scramble *v.* -shRNA1 cells post 4 Gy IR treatment. (**C**) Comparisons of the effect of elevated expression of AKR1C3 on proliferation of KY170R cells treated by doxorubicin. Figure S4, Analyses of AKR1C3 expression in 28 surgical samples of esophageal carcinomas. Among them 20 tumor samples are radioresistant and 8 are radiosensitive. Table S1, Characterizations of 28 esophageal cancer patients.
(RAR)
